# Decoding face recognition abilities in the human brain

**DOI:** 10.1093/pnasnexus/pgae095

**Published:** 2024-03-01

**Authors:** Simon Faghel-Soubeyrand, Meike Ramon, Eva Bamps, Matteo Zoia, Jessica Woodhams, Anne-Raphaelle Richoz, Roberto Caldara, Frédéric Gosselin, Ian Charest

**Affiliations:** Department of Experimental Psychology, University of Oxford, Oxford OX2 6GG, UK; Département de psychologie, Université de Montréal, Montréal, Québec H2V 2S9, Canada; Institute of Psychology, University of Lausanne, Lausanne CH-1015, Switzerland; Center for Contextual Psychiatry, Department of Neurosciences, KU Leuven, Leuven ON5, Belgium; Department for Biomedical Research, University of Bern, Bern 3008, Switzerland; Département de psychologie, Université de Montréal, Montréal, Québec H2V 2S9, Canada; School of Psychology, University of Birmingham, Hills Building, Edgbaston Park Rd, Birmingham B15 2TT, UK; Département de Psychology, Université de Fribourg, Fribourg CH-1700, Switzerland; Département de Psychology, Université de Fribourg, Fribourg CH-1700, Switzerland; Département de psychologie, Université de Montréal, Montréal, Québec H2V 2S9, Canada; Département de psychologie, Université de Montréal, Montréal, Québec H2V 2S9, Canada

## Abstract

Why are some individuals better at recognizing faces? Uncovering the neural mechanisms supporting face recognition ability has proven elusive. To tackle this challenge, we used a multimodal data-driven approach combining neuroimaging, computational modeling, and behavioral tests. We recorded the high-density electroencephalographic brain activity of individuals with extraordinary face recognition abilities—super-recognizers—and typical recognizers in response to diverse visual stimuli. Using multivariate pattern analyses, we decoded face recognition abilities from 1 s of brain activity with up to 80% accuracy. To better understand the mechanisms subtending this decoding, we compared representations in the brains of our participants with those in artificial neural network models of vision and semantics, as well as with those involved in human judgments of shape and meaning similarity. Compared to typical recognizers, we found stronger associations between early brain representations of super-recognizers and midlevel representations of vision models as well as shape similarity judgments. Moreover, we found stronger associations between late brain representations of super-recognizers and representations of the artificial semantic model as well as meaning similarity judgments. Overall, these results indicate that important individual variations in brain processing, including neural computations extending beyond purely visual processes, support differences in face recognition abilities. They provide the first empirical evidence for an association between semantic computations and face recognition abilities. We believe that such multimodal data-driven approaches will likely play a critical role in further revealing the complex nature of idiosyncratic face recognition in the human brain.

**Significance Statement**

The ability to robustly recognise faces is crucial to our success as social beings. Yet, we still know very little about the brain mechanisms allowing some individuals to excel at face recognition. This study builds on a sizeable neural dataset measuring the brain activity of individuals with extraordinary face recognition abilities—super-recognizers—to tackle this challenge. Using state-of-the-art computational methods, we show robust prediction of face recognition abilities in single individuals from a mere second of brain activity and reveal specific brain computations supporting individual differences in face recognition ability. Doing so, we provide direct empirical evidence for an association between semantic computations and face recognition abilities in the human brain—a key component of prominent face recognition models.

## Introduction

The ability to robustly recognize the faces of our colleagues, friends, and family members is paramount to our success as social beings. Our brains complete this feat with apparent ease and speed, in a series of computations unfolding within tens of milliseconds in a wide brain network comprising the inferior occipital gyrus, the fusiform gyrus, the superior temporal sulcus, and more anterior areas such as the anterior temporal lobe (1–3). Accumulating neuropsychological and behavioral evidence indicates that not all individuals, however, are equally competent at recognizing faces in their surroundings (4). Developmental prosopagnosics show a great difficulty at this task despite an absence of brain injury (5). In contrast, super-recognizers exhibit remarkable abilities for processing facial identity and can recognize individuals even after little exposure several years before (6–8). The specific nature of the neural processes responsible for these individual differences remains largely unknown. So far, individual differences studies have used univariate techniques to investigate face-specific aspects of brain processing. This revealed that contrasts between responses to faces compared to nonfaces, measured by the N170 event-related potential component or by the blood oxygen level dependent signals in regions of interest, are modulated by ability (9–15). However, univariate and contrast approaches are limited in their capacity to reveal the precise nature of the underlying brain computations (16–19).

**Fig. 1.**
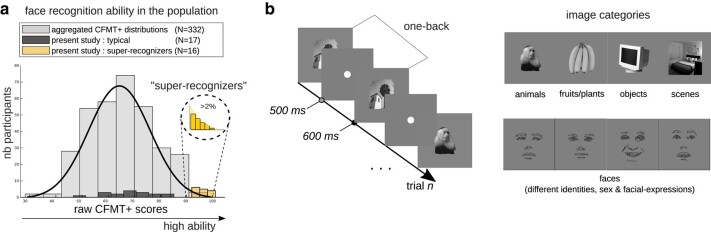
Experimental procedure. a) The histogram shows the Cambridge Face Memory Test long form (CFMT+ (8)) scores of super-recognizers (yellow bars), typical recognizers (black bars), and an additional 332 neurotypical observers from three independent studies for comparison (24–26). b) Participants engaged in a one-back task while their brain activity was recorded with high-density electroencephalography. The objects depicted in the stimuli belonged to various categories, such as faces, objects, and scenes. Note that the face drawings shown here are an anonymized substitute to the experimental face stimuli presented to our participants.

Here, we tackled this challenge with a data-driven approach. We examined the functional differences between the brains of super-recognizers and typical recognizers using decoding and representational similarity analyses (RSAs (18, 20–23)) applied to high-density electrophysiological (EEG) signals and artificial neural network models. We recruited 33 participants, including 16 super-recognizers, i.e. individuals better than the 98th percentile on a battery of face recognition tests (8) (Fig. 1a). We measured EEG in more than 100,000 trials while participants performed a one-back task. The objects depicted in the stimuli belonged to multiple visual categories including face images of different sexes, emotions, and identities, as well as images of man-made and nonface natural objects (e.g. a computer, a plant), animals (e.g. a giraffe, a monkey), and scenes (e.g. a city, a dining room; Fig. 1b).

## Results

### Behavioral results

All participants’ face recognition ability was assessed using the Cambridge Face Memory Test long form (CFMT+ (8)). Scores on the CFMT+ ranged from 50 to 85 in the typical recognizers group (M_TRs_ = 70.00; SD = 9.09), and from 92 to 100 in the experimental super-recognizers group (M_SRs_ = 95.38, SD = 2.68; difference between groups: *t*(31) = 10.6958, *P* < 0.00001 see Fig. 1a). The main experimental task was a one-back task (Fig. 1b). Accuracy was significantly greater for the super-recognizers (M_SRs_ = 0.8649, SD = 0.0626) than for the typical recognizers (M_TRs_ = 0.7591, SD = 0.096; *t*(30) = 3.6131, *P* = 0.0011). This was also true when analyzing separately face (M_SRs_ = 0.8677, SD = 0.0590; M_TRs_ = 0.7385, SD = 0.1048; *t*(30) = 4.2180, *P* = 0.00020) and nonface trials (M_SRs_ = 0.8619, SD = 0.0750; M_TRs_ = 0.7798, SD = 0.1000; *t*(30) = 2.6000, *P* = 0.0143). Furthermore, accuracy in the one-back task was positively correlated with scores on the CFMT+ (*r* = 0.68, *P* < 0.001; response time was marginally associated with CFMT+, *r* = 0.37, *P* = 0.04). We observed a significant difference in response times between the two groups for face stimuli (M_SRs_ = 0.6222 ms, SD = 0.1386 ms; M_TRs_ = 0.6817 ms, SD = 0.0660 ms; *P* = 0.0258) but not for nonface stimuli (M_SRs_ = 0.6262 ms, SD = 0.1401 ms; M_TRs_ = 0.6739 ms, SD = 0.0643 ms; *P* = 0.0801).

### Discriminating super-recognizers and typical recognizers from 1 s of brain activity

**Fig. 2.**
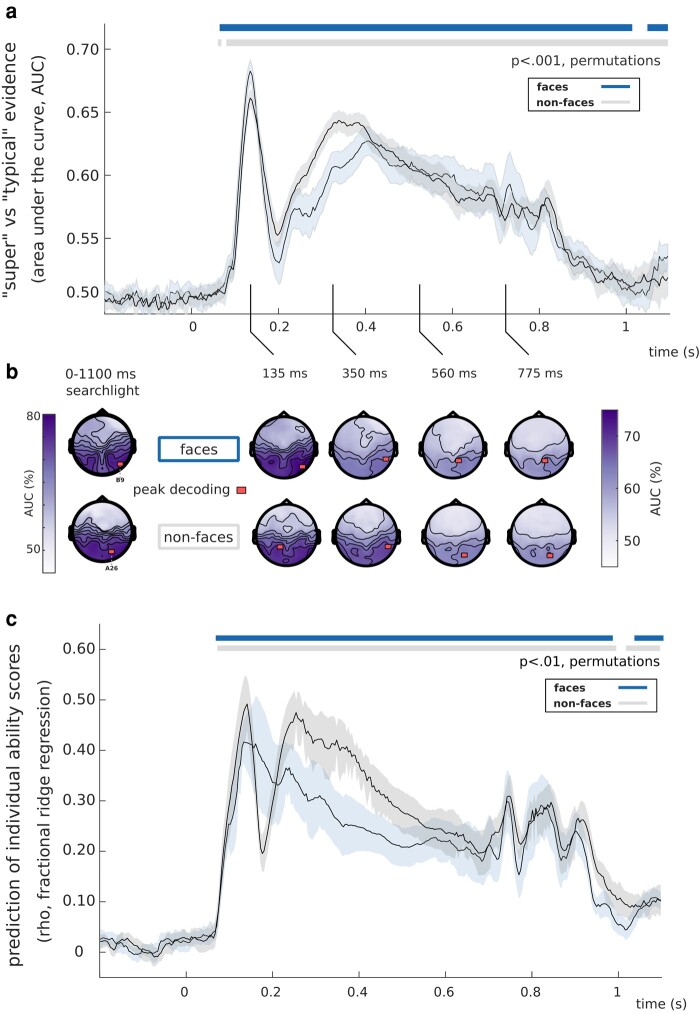
Decoding interindividual recognition ability variations from EEG activity. a) Trial-by-trial group-membership predictions (super-recognizer or typical recognizer) were computed from EEG patterns, for each 4-ms interval, while participants processed face (blue trace) or nonface stimuli (gray trace). Significant decoding performance occurred as early as 65 ms, peaked in the N170 window, and lasted for the remainder of the EEG epochs (*P* < 0.001). b) Topographies were obtained using searchlight decoding analyses, either concatenating all time points (left topographies) or for selected time windows (right topographies). Concatenating all time points resulted in peak classification performance of 77.3% over right occipitotemporal electrodes for face and 77.5% over right occipitotemporal electrodes for nonface conditions. In the N170 window, we observed a peak classification performance of 74.8% over right-temporal electrodes for face, and 72.1% over left-temporal electrodes for nonface conditions. c) We decoded the CFMT+ scores of the typical recognizers using fractional ridge regression. This yielded similar results with significant decoding as early as 75 ms, peaking around the N170 time window (peak-rho_face_ = 0.4149, peak-rho_nonface_ = 0.4899), and lasted for the remainder of the EEG epochs (*P* < 0.01, 1K permutations, 10 repetitions).

With this sizable and category-rich dataset, we first attempted to classify a participant as either a super- or a typical recognizer based solely on their brain activity. More specifically, we trained Fisher linear discriminants to predict group membership from single, 1-s trials of EEG patterns (in a moving searchlight of five neighboring electrodes; Fig. 2b). We observed up to ∼80% cross-validated decoding performance, peaking over electrodes in the right hemisphere. This performance is impressive given that the noise ceiling imposed on our classification by the test–retest reliability of the CFMT+ (8, 27), the gold-standard test used to identify super-recognizer individuals, is ∼93% (SD = 2.28%; see methods). To reveal the time course of these functional differences, we applied the same decoding procedure to each 4-ms interval of EEG recordings. Group-membership predictions attained statistical significance (*P* < 0.001, permutation tests, Fig. 2a) from about 65 ms to at least 1 s after stimulus onset, peaking around 135 ms, within the N170 window (28, 29).

Notably, similar results were obtained following the presentation of both face *and* nonface visual stimuli (Fig. 2a; see also Fig. S1). This did not result from face representations stored in short-term memory from one-back trials. Indeed, we repeated our decoding analysis for nonface trials either preceded by face trials or by nonface trials, and found significant decoding of group membership in both cases (Fig. S1). In addition, we successfully cross-decoded group membership from a model trained on face EEG activity and applied to nonface EEG activity (see Fig. S2). The decoding of group membership could be based on various features. Some of these features are directly related to brain representations for object and face recognition, and these aspects are further explored in subsequent sections of this article. On the other hand, there could be additional contributing features that are not directly linked to object and face recognition. For instance, differences in motor responses between the two subject groups might explain these results to some extent. However, excluding the 10% of trials with a motor response did not affect decoding accuracy (Fig. S2). Additionally, the decoding model might have relied on potential noise differences between the two subject groups. Nevertheless, our analysis did not reveal any evidence supporting such differences in the cross-participant similarity of the representational dissimilarity matrices (RDMs) for both groups (see the Linking neural representations with computational models of vision section; Fig. S3).

### Predicting individual recognition ability from 1 s of brain activity

An ongoing debate in individual differences research is whether the observed effects emerge from qualitative or quantitative changes in the supporting brain mechanisms (30–38). The decoding results presented up to this point might give the impression that face recognition ability is supported by qualitative differences in brain mechanisms. However, these results were obtained with dichotomous classification models applied, by design, to the brains of individuals from a bimodal distribution of ability scores (35).

To better assess the nature of the relationship between neural representations and ability in the general population, we thus performed a decoding analysis on the typical recognizers only, using a continuous regression model. Specifically, we used cross-validated fractional ridge regression (39) to predict *individual* CFMT+ face recognition ability scores from single-trial EEG data. This showed essentially similar results to the previous dichotomic decoding results: performance was above statistical threshold (*P* < 0.01, False Discovery Rate corrected) from about 80 ms to at least 1 s, peaking around 135 ms following stimulus onset for both face and nonface stimuli (Fig. 2c, peak-rho_face_ = 0.4149 at 133 ms, peak-rho_nonface_ = 0.4899 at 141 ms). This accurate decoding of individual scores from EEG patterns is compatible with a quantitative account of variations in brain mechanisms across individuals differing in face recognition abilities. Altogether, these decoding results provide evidence for important, quantitative and temporally extended variations in the brain activity supporting face recognition abilities. This extended decoding suggests effects of individual ability across multiple successive processing stages.

### Linking neural representations with computational models of vision

Decoding time courses, however, offer limited insights on the level of brain computations (40, 41). To better characterize the visual brain computations covarying with face recognition ability, we compared, using representational similarity analysis (20–22, 42), the brain representations of our participants to that of convolutional neural networks (CNNs) trained to categorize objects (43–45). These CNNs process visual features of gradually higher complexity and abstraction along their layers (45), from low-level (e.g. orientation, edges) to high-level features (e.g. objects and object parts).

**Fig. 3.**
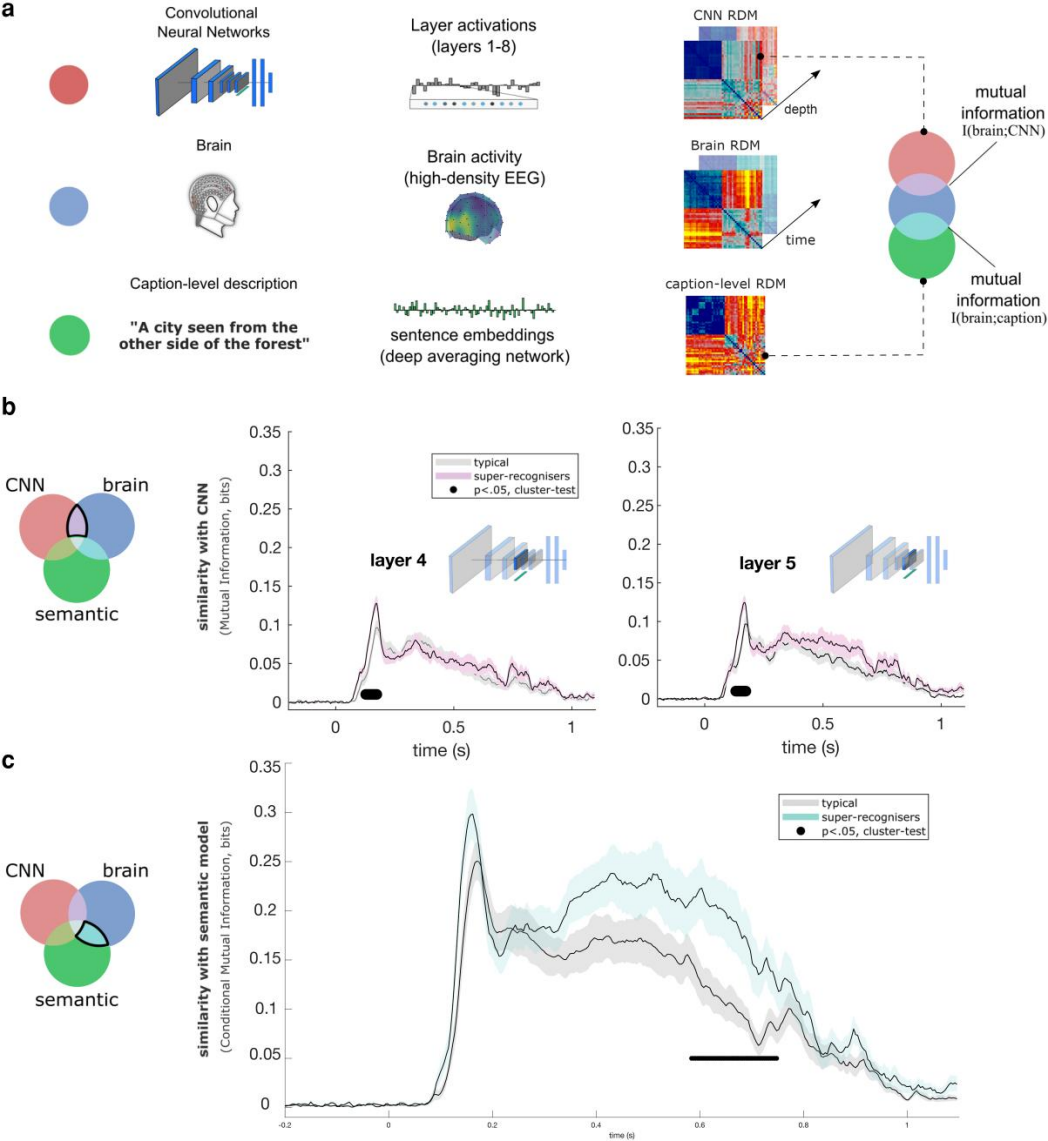
Comparison of super- and typical-recognizer brain representations with those of artificial neural networks of visual and semantic processing. a) RDMs were computed from CNNs (43, 44) of vision, human brain activity, and a deep neural network of caption classification and sentence semantics (50). To characterize the CNN RDMs, we computed the pairwise similarity between unit activation patterns for each image independently in each CNN layer. The caption-level RDMs were derived from human caption descriptions of the images transformed into sentence embeddings. Brain RDMs were computed using cross-validated decoding performance between the EEG topographies from each pair of stimuli at every 4 ms time point. Mutual information (48) between the model RDMs and the brain RDMs was assessed, for every participant, at each 4 ms step from stimulus onset. b) Mutual information between brain RDMs and AlexNet RDMs (removing shared mutual information between brain and semantic model) is shown for typical- (gray solid curve) and super-recognizers (pink solid curve). We found greater similarity with midlevel visual representations (layers 4 and 5 shown, but similar results were found for midlayers of VGG16, another popular CNN model; see Fig. S5) in the brains of super-recognizers (black line indicates significant contrasts, *P* < 0.05, cluster-corrected) between 133 and 165 ms. Similar results were observed when comparing brains and CNN models without removing the shared mutual information between brains and the semantic (caption-level) model (Fig. S5). c) Mutual information with the semantic model (excluding shared mutual information between brain and AlexNet) differed for typical- and super-recognizers in a later time window centered around 650 ms (cyan curve; super > typical, *P* < 0.05, cluster-corrected). Again, similar results were observed when comparing brains and the semantic model without removing the shared mutual information between the brain and CNN model (see Fig. S8).

The brain representations were characterized by computing RDMs for each participant and for each 4-ms time interval. These brain RDMs were derived using the cross-validated decoding performance of a linear discriminant model, where brain activity was decoded for every pair of stimuli at a given time interval (46, 47); see Fig. S4 for the group-average RDMs and time course of key categorical distinctions. The visual model representations were characterized by computing RDMs from the layers of the CNNs, using Pearson correlations of the unit activations across all pairs of stimuli. Compared to typical participants, we found that the brain RDMs of super-recognizers showed larger mutual information (48) with the layer RDMs of CNNs that represent midlevel features (e.g. combinations of edges, contour, shape, texture (45, 49)) between 133 and 165 ms (Fig. 3a, *P* < 0.05, cluster-test; see also Fig. S5 for similar results with unconstrained analyses; supplementary analyses on specific category conditions in the RDMs are shown in Fig. S6). These results indicate that midlevel representations of an object-trained CNN matched the representations of super-recognizers more closely than those of typical participants. We replicated these results using a face-trained CNN, e.g. (51–53), VGGface (54), which possess midlevel representations similar to those of object-trained CNN (see Fig. S7; *P* < 0.05, cluster-test). The stronger association between brain representational geometries of the super-recognizers with computational models of vision could be explained by a marked difference in signal to noise between the two groups of participants. To control for this potential confound, we computed the cross-participant similarity of the RDMs in both groups (see Fig. S3). If the signal to noise was larger in the super-recognizers, we would expect larger cross-participant similarity of the RDMs; however, we observed no significant difference between the two groups.

### Linking neural representations with computational model of semantics

The finding that ability decoding was significant as late as 1 s after stimulus onset hints that brain computations beyond what is typically construed as pure visual processing also differ as a function of face recognition ability. To test this hypothesis, we asked five new participants to write captions describing the images presented during our experiment (e.g. “A city seen through a forest.”), and used a deep averaging network (Google Universal Sentence Encoder, GUSE (50)) to transform these captions into embeddings (points in a caption space). GUSE has been trained to predict semantic textual similarity from human judgments, and its embeddings generalize to an array of other semantic judgment tasks (50). We then compared the RDMs computed from this semantic model to the brain RDMs of both typical- and super-recognizers. Importantly, both this comparison, and the one comparing brain and visual models, excluded the information shared between the semantic and visual models (but see Fig. S8 for similar results with unconstrained analyses). We found larger mutual information with these semantic representations in the brains of super-recognizers than in those of typical recognizers in a late window between 598 and 727 ms (Fig. 3c, *P* < 0.05, cluster-test). Supplementary analyses on specific stimulus categories of the RDMs (Fig. S9) suggest that these results emerged mainly from the face vs. face and face vs. nonface stimuli pair conditions.

### Linking neural representations with behavioral representations for shape and semantic similarity judgments

**Fig. 4.**
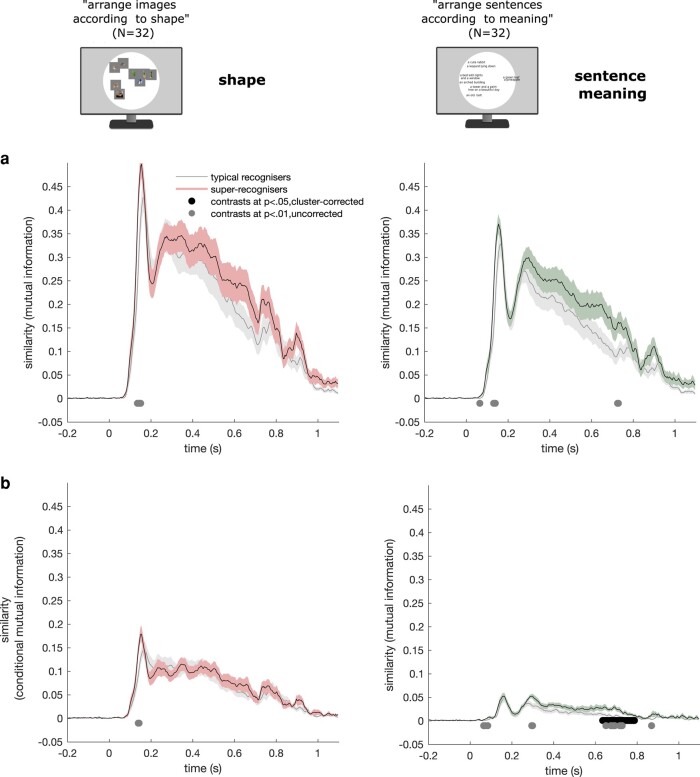
Linking neural representations with behavioral representations for shape, function, and semantic similarity judgments. a) Mutual information between brain RDMs and the mean RDM built from shape similarity judgments (first column) and sentence meaning similarity judgments (second column) is shown for typical- (gray curves) and super-recognizers (coloured curves). Greater similarity with shape information in the brains of super-recognizers (*P* < 0.01, uncorrected; *P*-corrected_MI_ = 0.1259) and greater similarity with sentence meaning information for super-recognizers (*P* < 0.01, uncorrected; *P*-corrected_MI_ = 0.0819) only reached significance before cluster corrections. The shaded areas of all curves represent the SE. b) Conditional mutual information between brain RDMs and the mean RDM built from similarity judgments of shape and of sentence caption meaning (removing shared mutual information between brain and sentence caption meaning RDM for shape similarity, and vice versa) is shown in typical- and super-recognizers. We found greater similarity with sentence meaning in the brains of super-recognizers between 635 and 787 ms (black line indicates significant contrasts, *P* < 0.05, cluster-corrected), in agreement with our comparisons with the artificial semantic model (Fig. 3c). Greater similarity with shape information in the brains of super-recognizers only reached significance before cluster corrections (*P* < 01, uncorrected; *P*-corrected_CMI_  *=* 0.2098).

Our findings so far suggest that midlevel visual and semantic brain processes both support individual differences in face recognition abilities. We looked for further support for these conclusions using RDMs derived from a behavioral experiment. A group of 32 new human participants were submitted to two multiple arrangement tasks (55–57) in which they were asked to evaluate the shape similarities of all pairs of the 49 visual stimuli used in the main experiment, and the meaning similarities of all pairs of the 49 mean sentence captions produced by five human participants to describe these images and used for the semantic model (see the Linking neural representations with computational model of semantics section). More specifically, participants arranged the images/sentences inside a white circular arena according to the task instructions using simple drag and drop operations (see Fig. 4). We computed the mutual information between the mean RDMs extracted from each of these tasks and the time-resolved brain RDMs of super- and typical recognizers as well as the same while excluding the information shared with the other task. Results indicated only a trend for shape representations being enhanced around midlatencies in super-recognizers relative to typical recognizers which did not survive cluster correction (*P* < 0.01, uncorrected; *P*_MI_ = 0.1259; *P*_CMI_ = 0.2098; cluster-corrected; see Fig. 4). Meaning representations were enhanced in late latencies in super-recognizers compared to typical recognizers (sentence meaning: 635–787 ms, *P* < 0.05, cluster-corrected; see Fig. 4). These results confirm that semantic representations at relatively late latencies and, to a lesser degree, shape representations at midlatencies are enhanced in the brains of super-recognizers.

## Discussion

Using a data-driven approach combining neuroimaging, computational models, and behavioral tests, we characterized the computations modulated by variations in face recognition ability in the human brain. We recorded high-density electroencephalographic (EEG) responses to face and nonface stimuli in super-recognizers and typical recognizers. Using multivariate analysis, we reliably decoded group membership as well as recognition abilities of single individuals from a single second of brain activity. We then characterized the neural computations underlying these individual differences by comparing human brain activity with representations from artificial neural network models of vision and semantics using representational similarity analysis. Furthermore, we compared the brain activity with similarity judgments derived from additional human participants engaged in two tasks. In the first task, participants judged our visual stimuli on the similarity of their shape, while in the second task, participants judged sentence captions describing these stimuli on the similarity of their meaning. These sets of comparisons revealed two main findings. First, we found higher similarity between early brain representations of super-recognizers and midlevel representations of vision models as well as, to a lesser degree, shape similarity judgments. Second, this approach revealed higher similarity between late brain representations of super-recognizers and representations of an artificial semantic model as well as sentence caption similarity judgments. To our knowledge, this is the first demonstration of a link between face recognition ability and brain computations beyond high-level vision. Overall, these findings revealed specific computations supporting our individual ability to recognize faces and suggest widespread variations in brain processes related to this crucial ability.

We achieved robust decoding of face recognition ability when examining EEG responses to face *and* nonface stimuli. This is consistent with several neuropsychological (33, 58–62) and brain imaging findings (12, 32, 63, 64) showing face and nonface processing effects in individuals across the spectrum of face recognition ability (19, 65), but see Refs. (13, 66–68).

The decoding we observed for face and nonface stimuli peaked at right occipitotemporal electrodes, in the temporal window around the N170 component (28). At that time, the representations in the brains of our participants differed most with respect to the mid-layer representations of artificial models of vision. These layers have been previously linked to processing in human inferotemporal cortex (45, 69–71) and functionally to midlevel feature representations such as combinations of edges and parts of objects (45, 49). Such associations with the N170, however, do not mean that this component is exclusively involved in these midlevel processes. Rather, it suggests that other visual computations, including the high-level visual computations usually associated with the N170, do not differ substantially between super-recognizers and typical recognizers. The fact that these midlevel features are mostly shared between face and nonface stimuli could explain at least partly the high decoding performance observed for both classes of stimuli. They suggest that a midlevel visual processing is enhanced in super-recognizers leading to improved processing of faces and objects.

Crucially, we found that face recognition ability is also associated with semantic computations that extend beyond basic-level visual categorization in a late time window around the P600 component (72–74). Recent studies using computational techniques have shown that word representations derived from models of natural language processing explain significant variance in the visual ventral stream (18, 75–77). The current study goes beyond this recent work in two ways. First, our use of human sentence description and sentence encoders to characterise semantic (caption-level) computations provides a more abstract description of brain representations. Second, and most importantly, our work revealed a link between semantic brain computations and individual differences in face recognition ability. An association between semantic processes and face recognition ability had been posited in models of face recognition (1, 78) but, to our knowledge, it had never been shown empirically before.

Overall, thus, our findings suggest important differences in perceptual and semantic representations in individuals with outstanding ability to recognize faces. The higher similarity with computational models of vision indicates that super-recognizers have more efficient midlevel representations. These enhanced representations suggest that part-based information about faces and objects, putatively emerging from midlevel occipitotemporal regions (79), is richer in individuals with strong face recognition abilities (24). Furthermore, our findings show that the more similar the brain representations of an individual are to task-optimized computational models of semantics, the better they are at recognizing faces. These enhanced late semantic representations, for example, might emerge from enhanced subordinate-level information about objects and faces (80, 81).

Our approach of decoding group membership to reveal electrophysiological differences between super-recognizers and typical recognizers, followed by representational similarity analysis with computational models of vision and language, enabled revealing potential mechanisms underlying enhanced recognition ability. Other possible differences between our participants might also contribute to our ability to decode their brain signals. Differences in top-down (i.e. attention) mechanisms, better ability to memorise images more generally, or both, could also lead to enhanced representations. Interestingly, we could decode from both face *and* nonface categories, and across categories (training with face trials and testing on nonface trials) suggesting that the mechanisms subtending the enhanced abilities of super-recognizers are not restricted to faces (4, 59).

Furthermore, while our decoding approach indicates that important differences in brain processing emerge from early (80 ms) to late (∼1 s) processing windows, our RSA modeling approach only explained part of these processing windows. Specifically, while broad visual (∼150 ms) and semantic representations (∼600 ms) were found to differ in super-recognizers using this computational approach, it still remains to be shown what specific representations are critical in differentiating the best face recognizers during other windows of processing (e.g. mid-late processing around 400 ms).

## Conclusion

Our results offer a stepping stone for a better understanding of face recognition idiosyncrasies in the human brain. Indeed, with the development of novel and better artificial models simulating an increasing variety of cognitive processes, and with the technological advances allowing the processing of increasingly larger neuroimaging datasets, the approach described here provides a new and promising way to tackle the link between individual differences in human behavior and specific computations in the brain. In addition, this decoding approach may provide quick and accurate alternatives to standardized behavioral tests assessing face recognition ability, for example in the context of security settings that benefit from strong face processing skills among their personnel (such as police agencies, border patrol, etc.). It could also be used in a closed-loop training procedure designed to improve face recognition ability (82).

## Methods

### Participants

A total of 33 participants were recruited for this study. The first group consisted of 16 individuals with exceptional ability in face recognition—super-recognizers. The second group was composed of 17 neurotypical controls. These sample sizes were chosen according to the effect sizes described in previous multivariate object recognition studies (46, 47, 57). The data from one super-recognizer were excluded due to faulty EEG recordings. No participant had a history of psychiatric diagnostic or neurological disorder. All had normal or corrected to normal vision. This study was approved by the Ethics and Research Committee of the University of Birmingham, and informed consent was obtained from all participants.

Sixteen previously known super-recognizers were tested in the current study (30–44 years old, 10 female). Eight of these (SR1–SR8) were identified by Prof. Josh P. Davis from the University of Greenwich using an online test battery comprising a total of six face cognition tasks (6) and tested at the University of Birmingham. The remaining eight (SR9 to SR16) were identified using three challenging face cognition tests (7) and were tested at the University of Fribourg. The behavioral test scores for all participants are provided in Tables S1 and S2. Across SR cohorts, the Cambridge Face Memory Test long form (CFMT+ (8)) was used as the measure of face identity processing ability. A score greater than 90 (i.e. 2 SD above average) is typically considered the threshold for super-recognition (8, 59, 83). Our 16 super-recognizers all scored above 92 (*M* = 95.31; SD = 2.68). A score of 92 corresponds to the 99th percentile according to our estimation from a group of 332 participants from the general population recruited in three independent studies (24–26).

An additional 17 typical recognizers (20–37 years old, 11 female) were recruited and tested on campus at the University of Fribourg (*n* = 10) and the University of Birmingham (*n* = 7). Their CFMT+ scores ranged from 50 to 85 (*M* = 70.00; SD = 9.08). Neither the average nor the distribution of this sample differed significantly from those of the 332 participants from the general population mentioned above (see Fig. 1a; *t*(346) = 1.3065, *P* = 0.1922; two-sample Kolmogorov–Smirnov test; *D*(346) = 0.2545, *P* = 0.2372).

### Tasks

#### CFMT+

All participants were administered the CFMT long form, or CFMT + (8). In the CFMT+, participants are required to memorize a series of face identities, and to subsequently identify the newly learned faces among three faces. It includes a total of 102 trials of increasing difficulty. The duration of this test is about 15 min. EEG was not recorded while participants completed this test.

#### One-back task

##### Stimuli

The stimuli used in this study consisted of 49 images of faces, animals (e.g. giraffe, monkey, puppy), plants, objects (e.g. car, computer monitor, flower, banana), and scenes (e.g. city landscape, kitchen, bedroom). The 24 faces (13 identities, 8 males, and 8 neutral, 8 happy, 8 fearful expressions) were sampled from the Radboud Face dataset (84). The main facial features were aligned across faces using Procrustes transformations. Each face image was revealed through an ellipsoid mask that excluded nonfacial cues. The nonface images were sampled from the stimulus set of Kiani et al. (85). All stimuli were converted to 250 × 250 pixels (8 × 8° of visual angle) grayscale images. The mean luminance and the luminance SD of these stimuli were equalized using the SHINE toolbox (86).

##### Procedure

We measured high-density electroencephalographic (EEG; sampling rate = 1,024 Hz; 128-channel BioSemi ActiveTwo headset) activity while participants performed ∼3,200 trials of a one-back task in two recording sessions separated by at least one day and by a maximum of 2 weeks (Fig. 1b). Participants were asked to press a computer keyboard key on trials where the current image was identical to the previous one. Repetitions occurred with a 0.1 probability. They were asked to respond as quickly and accurately as possible. Feedback was done in the form of a change in color of the fixation point (red or green) after a repetition trial (which happened on a 0.1 probability basis). This was done to help participants pay attention during the task. Target trials were not excluded from the analyses. A trial unravelled as follows: a white fixation point was presented on a gray background for 500 ms (with a jitter of ± 50 ms); followed by a stimulus presented on a gray background for 600 ms; and, finally, by a white fixation point on a gray background for 500 ms. Participants had a maximum of 1,100 ms following stimulus onset to respond. This interval, as well as the 200 ms preceding stimulus onset, constituted the epoch selected for our EEG analyses. In total, our participants completed 105,600 one-back trials which constituted ∼32 h of EEG epochs.

#### Shape and sentence meaning multiple arrangement tasks

Thirty-two new neurotypical participants took part in two multiple arrangement tasks (22, 87) in counterbalanced orders. In two of the tasks, they were asked to evaluate the shape or function similarities of the 49 stimuli used in the main experiment while, in the other task, they were instructed to judge the meaning similarities of sentence captions describing these stimuli (see the Semantic caption-level deep averaging neural network RDM section for more information about these sentence captions).

More specifically, participants were asked to arrange stimuli or sentence captions on a computer screen inside a white circular arena by using computer mouse drag and drop operations. During the shape/function (vs. meaning) multiple arrangement task, they were instructed to place the displayed visual stimuli (vs. sentence captions) in such a way that their pairwise distances match their shape/function (vs. meaning) similarities as much as possible (Fig. 4). On the first trial of each task, participants arranged all 49 items. On subsequent trials, a subset of these items was selected based on an adaptive procedure aimed at minimizing uncertainty for all possible pairs of items (e.g. items that initially were placed very close to each other) and at better approximating the high-dimensional perceptual representational space (87). This procedure was repeated until the task timed out (20 min).

We computed one representational dissimilarity matrix (RDM) per task per participant. Three participants were excluded from the final sample because their RDMs differed from the mean RDMs by more than 2 SDs. Finally, we averaged the remaining individual RDMs within each task.

## Analyses

All reported analyses were performed independently for each EEG recording session and then averaged. Analyses were completed using custom code written in MATLAB (MathWorks) and Python.

### EEG preprocessing

EEG data were preprocessed using FieldTrip (88): continuous raw data were first rereferenced relative to Cz, filtered with a band-pass filter (0.01–80 Hz), segmented into trial epochs from −200 to 1,100 ms relative to stimulus onset, and down-sampled at 256 Hz.

### Decoding analyses

#### Whole-brain analyses

To predict group membership from EEG brain activity, we trained Fisher linear discriminant classifiers to predict participants’ group membership based on raw EEG topographies, using all 128 channels of single-trial EEG data as features. Notably, here, the decoding is made on an *across-participants* basis. This was done across all trials of either face or nonface condition, for each of the two sessions separately (∼26,000 observations per condition, per session, 5-fold cross-validation, 5 repetitions (89, 90)). The number of trials was matched across participants. This process was repeated over all EEG time samples separately, starting from −200 ms and ending to 1,100 ms after stimulus onset, creating decoding accuracy time courses. The area under the curve (AUC) was used to assess sensitivity. Decoding time courses were averaged across the two EEG sessions. The resulting evidence indicates when super-recognizers can be categorized from brain activity when processing faces (blue) and nonface stimuli (gray), as shown in Fig. 2a. Additional control decoding analyses investigating effects of one-back trials on the predictions are shown in Fig. S1. These trials required that our participant compared their representations of the presented image and the one stored in short-term memory. This showed similar findings, with one notable difference being that the face–face discrimination condition was the one that obtained peak decoding accuracy.

#### Searchlight analysis

We conducted a searchlight analysis decoding EEG signals from all subsets of five neighboring channels to characterize the scalp topographies of group-membership AUC. This searchlight analysis was done either using the entire EEG time series of a trial (0–1,100 ms; Fig. 2b, leftmost topographies) or using 60 ms temporal windows (centered on 135, 350, 560, and 775 ms; Fig. 2b rightmost topographies). We ran additional control searchlight decoding procedures investigating the effect of one-back trials (Fig. S1).

#### Regression analysis

We used fractional ridge regression models (39) to predict individual face recognition ability scores (CFMT+) among the typical recognizers from EEG patterns across time. We trained our model on subsets of 60% of the EEG patterns. We chose the alpha hyperparameter with the best coefficient of determination among 20 alpha hyperparameters ranging linearly from 0.001 to 0.99 applied on a 30% validation set. The decoding performance was assessed using the Spearman correlation between the CFMT+ scores and predictions from the overall best model (applied on the remaining 10% of EEG patterns). This process was repeated 10 times and the Spearman correlations were averaged. Significance was assessed using a permutation test (see the Group comparison and inferential statistics section).

### Representational similarity analysis of brain and computational models

We compared our participants’ brain representations to those from visual and semantic (caption-level) artificial neural networks using RSA (20–22, 42).

#### Brain RDMs

For every participant, we trained a Fisher linear discriminant to distinguish pairs of stimuli from every 4-ms intervals of EEG response (on all 128 channels) to these stimuli from −200 to 1,100 ms after stimulus onset (91, 92). Cross-validated AUC served as pairwise classification dissimilarity metric. By repeating this process for all possible pairs (1,176 for our 49 stimuli), we obtained an RDM. RDMs are shown for selected time points in Fig. S4.

#### Visual convolutional neural network RDMs

We used a pretrained AlexNet (43) as one model of the visual computations along the ventral stream (45). Our 49 stimuli were input to AlexNet. Layerwise RDMs were constructed comparing the unit activation patterns for each pair of images using Pearson correlations. Similarly, we computed layerwise RDMs from another well-known CNN, VGG-16 (see Fig. S5). Following previous studies using this model (93, 94), we averaged the convolutional layer RDMs situated between each max pooling layers and the layers’ input into five aggregated convolutional RDMs (e.g. conv1-1 and conv1-2 into RDM-conv1); this facilitated the comparison of our results with the five convolutional layers of AlexNet.

#### Semantic caption-level deep averaging neural network RDM

We asked five new participants to provide a sentence caption describing each stimulus (e.g. “a city seen from the other side of the forest,” see Fig. 1d) using the Meadows online platform (www.meadows-research.com). The sentence captions were input in GUSE (50) resulting in 512-dimensional sentence embeddings for each stimulus. We then computed the dissimilarities (cosine distances) between the sentence embeddings across all pairs of captions, resulting in a semantic caption-level RDM for each participant. The average RDM was used for further analyses.

### Comparing brain representations with computational models

We compared our participants’ brain RDMs to those from the vision (Fig. 3b) and semantic (Fig. 3c) models described in the previous section using conditional mutual information (CMI) (48), which measures the statistical dependence between two variables (e.g. mutual information *I*(*x*; *y*)), removing the effect from a third variable (i.e. *I*(*x*; *y*|*z*)). Additional comparisons using unconstrained mutual information between brain RDMs and both models are shown in Figs. S5 and S8.

### Group comparison and inferential statistics

#### Comparison of CMI

Time courses of CMI were compared between the super-recognizers and typical recognizers using independent samples t tests and a Monte Carlo procedure at a *P-*value of 0.05, as implemented in the Fieldtrip Toolbox (88). Familywise errors were controlled for using cluster-based corrections, with maximum cluster size as cluster-level statistic and an arbitrary *t* threshold for cluster statistic of (−1.96, 1.96) for the comparison of brain and semantic (excluding CNN) and for the comparison of brain and CNN (excluding semantic) time courses. The SE is shown for all curves as color-shaded areas (Fig. 3). Analyses with mutual information (MI) (brain; CNN) and MI (brain; semantic) were completed in an identical manner.

#### Time course of group-membership decoding

Significance was assessed using nonparametric permutation tests. We simulated the null hypothesis by training the linear classifier to identify shuffled group-membership labels from the experimental EEG patterns. This process was repeated 1,000 times for each time point and each one of the two sessions. We then compared the real, experimental decoding value at each time point to its corresponding null distribution, and rejected the null hypothesis if the decoding value was greater than the prescribed critical value at a *P* < 0.001 level.

#### Time course of individual ability decoding using ridge regression

Significance was again assessed using nonparametric permutation testing. The ridge regression analysis predicted cross-validated CFMT+ scores from single-trial EEG patterns, and goodness of fit is reported using Spearman's correlation between the predicted and observed CFMT+ scores. Under the null hypothesis that all participants elicited comparable EEG response patterns, irrespective of their CFMT+ score, the face recognition ability scores are exchangeable. We simulated this null hypothesis by repeating the ridge regression model training using randomly shuffled CFMT+ scores. The predicted CFMT+ scores were then correlated to the empirical, observed CFMT+ scores using Spearman's correlation, and this was repeated 1,000 times for each time point. We finally compared the real, experimental correlation value with its corresponding null distribution at each time point, and rejected the null hypothesis if the correlation value was greater than the prescribed critical value at a *P* < 0.01 level.

## Supplementary Material

pgae095_Supplementary_Data

## Data Availability

High-density EEG data associated with this article are available on The Open Science Framework (https://osf.io/pky28/).

## Preprints

This manuscript was posted on a preprint: https://www.biorxiv.org/content/10.1101/2022.03.19.484245v3.
