# Supplementary material for: Decoding face recognition abilities in the human brain
Source: PNAS Nexus. 2024 Mar 1;3(3):pgae095. doi: 10.1093/pnasnexus/pgae095 (PMC10957238; doi:10.1093/pnasnexus/pgae095)
Supplement: pgae095_Supplementary_Data [file pgae095_supplementary_data.docx]

**Supplementary material**

**Scores obtained by the super-recogniser tested in the UK on a battery of face recognition tests**

| subject-ID | CFMT+ | GFMT | Face Array | LASIE Match | Black and White | Super-rec | Longterm | one-back  faces | one-back  non-faces |
| --- | --- | --- | --- | --- | --- | --- | --- | --- | --- |
| SR-1 | 93 | 40 | 38 | 83 | 34 | 13 | 9 | .9328 | 0.977 |
| SR-2 | 95 | 40 | 37 | 88 | 33 | 12 | 9 | .8500 | 0.7903 |
| SR-3 | 97 | 40 | 32 | 84 | 33 | 12 | 8 | .9325 | 0.9718 |
| SR-4 | 93 | 40 | 38 | 92 | 34 | 13 | 8 | .9726 | 0.9835 |
| SR-5 | 98 | 40 | 31 | 91 | 33 | 14 | 9 | .9326 | 0.9787 |
| SR-6 | 100 | 40 | 39 | 82 | 40 | 14 | 10 | .9823 | 0.9817 |
| SR-7 | 98 | 40 | 40 | 92 | 33 | 13 | 8 | .9319 | 0.9647 |
| SR-8 | 96 | 38 | 40 | 90 | 39 | 12 | 10 | .9362 | 0.9787 |
| Max score | 102 | 40 | 40 | 100 | 40 | 14 | 10 | 1 | 1 |

**Table S1:** The scores above show performance on a battery of standardised face recognition tests (Noyes et al.,2021) for the participants identified as super-recognisers in the UK. Also shown are the scores for our one-back task, for face and non-face trials. The last row of the table shows the maximum obtainable score for each test.

**Scores obtained by the super-recogniser tested in Switzerland on a battery of face recognition tests**

| subject-ID | CFMT+ | FICST score | YBT long raw score | one-back  faces | one-back  non-faces |
| --- | --- | --- | --- | --- | --- |
| SR-9 | 92 | 0 | 29 | .8132 | 0.8384 |
| SR-10 | 99 | 0 | 17 | .9409 | 0.9619 |
| SR-11 | 92 | 0 | 20 | .9635 | 0.9808 |
| SR-12 | 93 | 1 | 20 | .8581 | 0.8212 |
| SR-13 | 92 | 7 | 17 | .9530 | 0.985 |
| SR-14 | 96 | 0 | 18 | .9604 | 0.9886 |
| SR-15 | 94 | 3 | 16 | .9112 | 0.9624 |
| SR-16 | 97 | 1 | 22 | .9792 | 0.9643 |
| Best score | 102 | 0 | 35 | 1 | 1 |

**Table S2:** The scores above show performance on a battery of standardised face recognition tests (Ramon et al., 2021) for the participants identified as super-recognisers in Switzerland. Also shown are the scores for our one-back task, for face and non-face trials. The last row of the table shows the best obtainable score for each test.

**Univariate analyses of EEG associated with individual ability**

*N170 amplitude and latency.* We also performed more traditional event-related potential (ERP) analyses for both groups. We extracted, for face and non-face trials, peak negative ERP amplitudes and latencies for every participant in a window corresponding to the N170 component (within 110-200 ms at electrodes [B6, B7, B8, A28] on the right hemisphere and [A9, A10, A11, A15] on the left hemisphere). We tested the conditions, groups, and their interaction effects using an ANOVA on N170 peak latency and amplitude separately. No interaction effects were observed for peak latencies (F_interaction_(60,1)=1, p=.32) and amplitudes (F_interaction_(60,1)=0.32, p>.50). The peak N170 was earlier (F_conditions_(60,1)=5.86, p=.0185) and presented greater amplitudes (F_conditions_(60,1)=33.78, p<.0001) for faces compared to non-face objects. Moreover, the peak N170 was earlier (F_group_(60,1)=19.23, p<.0001) and presented larger amplitudes (F_group_(60,1)=13.75, p=.0005) in super-recognisers than typical recognisers.

*Lateralisation.* Compared to typical recognisers, super-recognisers showed greater N170 peak amplitudes in the right-hemisphere electrodes for faces (computed as the difference between the right [B6, B7, B8, A28] and left [A9, A10, A11, A15]; t(30)=-2.8542, p=.01). Moreover, the CFMT+ scores of typical recognisers correlated with right-hemisphere lateralisation of the N170 for faces (r(16)=-.53, p=.0298). These effects were not significant either for non-face stimuli, or for N170 peak latency.

**
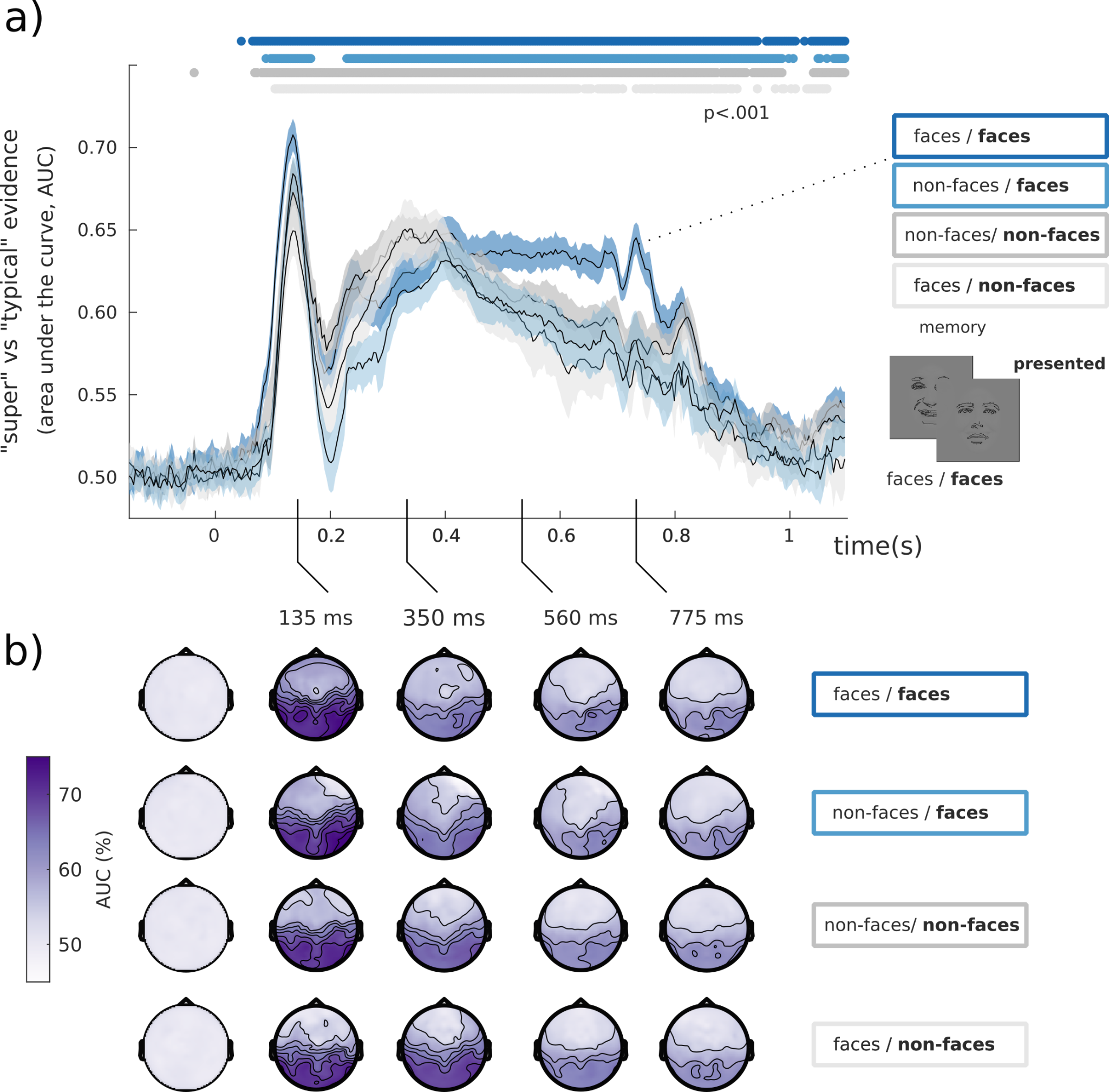
**

**Figure S1**. **a)** We computed the time course of decoding accuracy for group membership from all different face and non-face combinations of one-back and current trials (e.g. consecutive face - face trials). We observed a similar time course for all combinations. However the consecutive face-face trials showed larger decoding accuracies around 400 to 750 ms. **b)** The topographies show results from a searchlight decoding analysis with classification performance attaining 75% accuracy around 135 ms over occipito-temporal electrodes for face presented conditions (74.6% for face-face, 75.1% for nonface-face) and 72% for non-face presented conditions (71.7% for nonface-nonface, 71.5% for face-non-face). Note that drawings of faces are depicted here as an anonymised substitute to the experimental face stimuli presented to our participants.

**
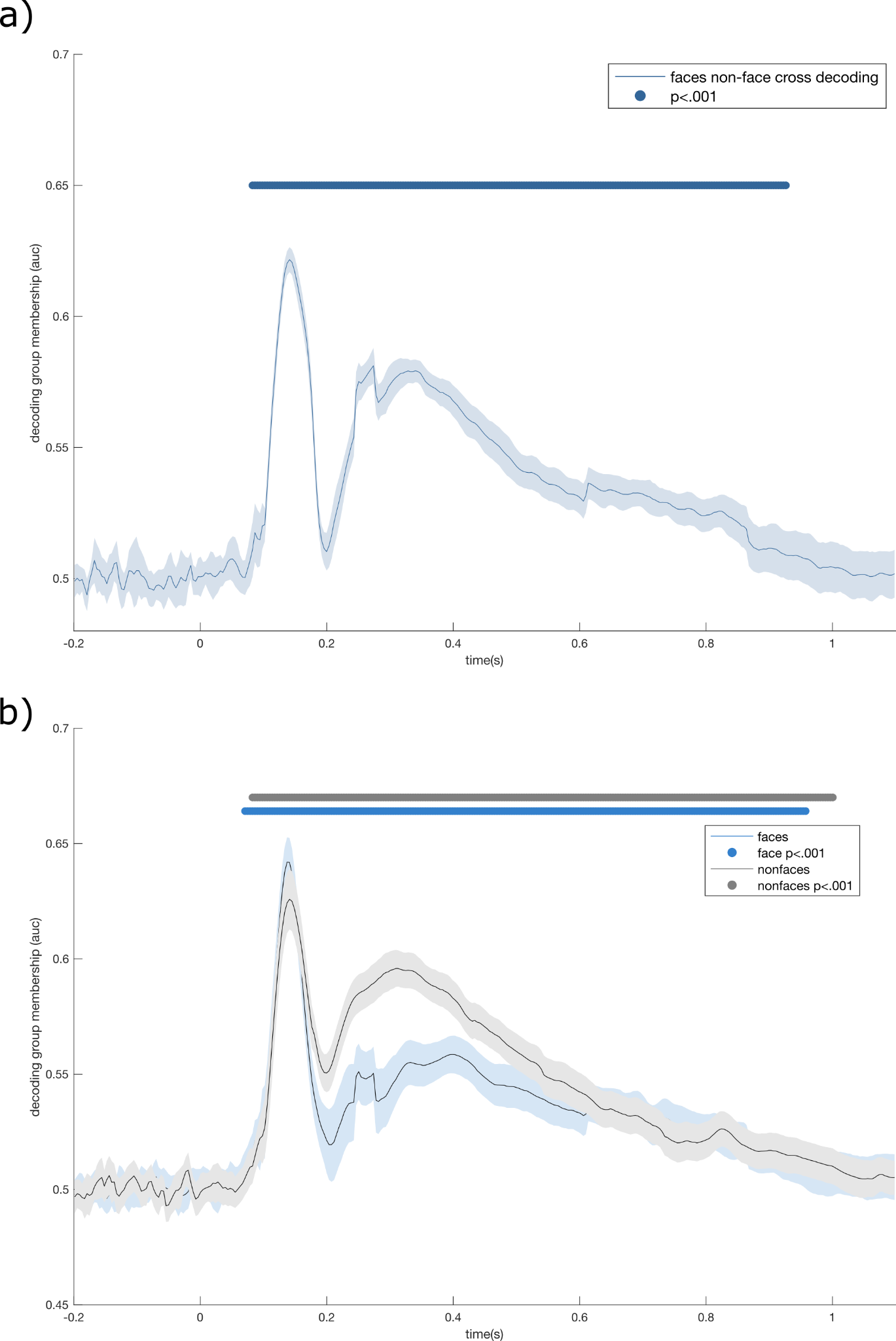
**

**Figure S2. a)** We computed the time course of decoding AUC for group membership by cross-decoding from face to non-face conditions, also excluding trials with motor responses. This still showed significant cross-decoding (83 ms - 930 ms; *p* <.001, permutations). **b)** We computed the time course of decoding AUC for group membership from face and non-face conditions, excluding trials with motor responses. This showed similar results for both decoding from faces (70 ms - 958 ms; *p*<.001, permutations) and non-faces stimuli (83 ms - 1000 ms; *p* <.001, permutations).


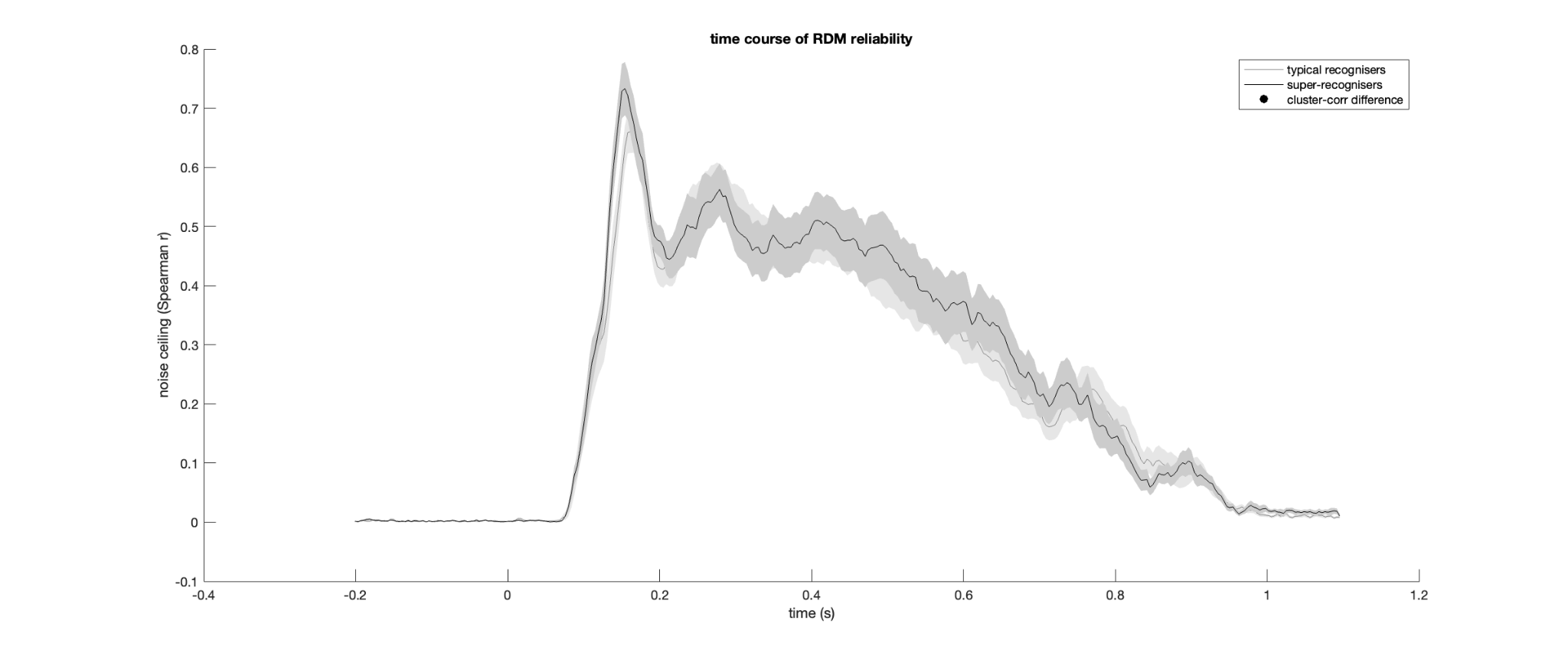


**Figure S3.** We computed the cross-participant similarity in the RDMs of both groups. Overall, the contrasts between SRs and TRs showed no differences between SRs and TRs at any latencies, thus excluding the “better signal-to-noise-ratio” interpretation of our results, and concluding that the mid and late processing effects found in SRs vs. TRs emerge from genuine change in the representational geometry of SRs.

**
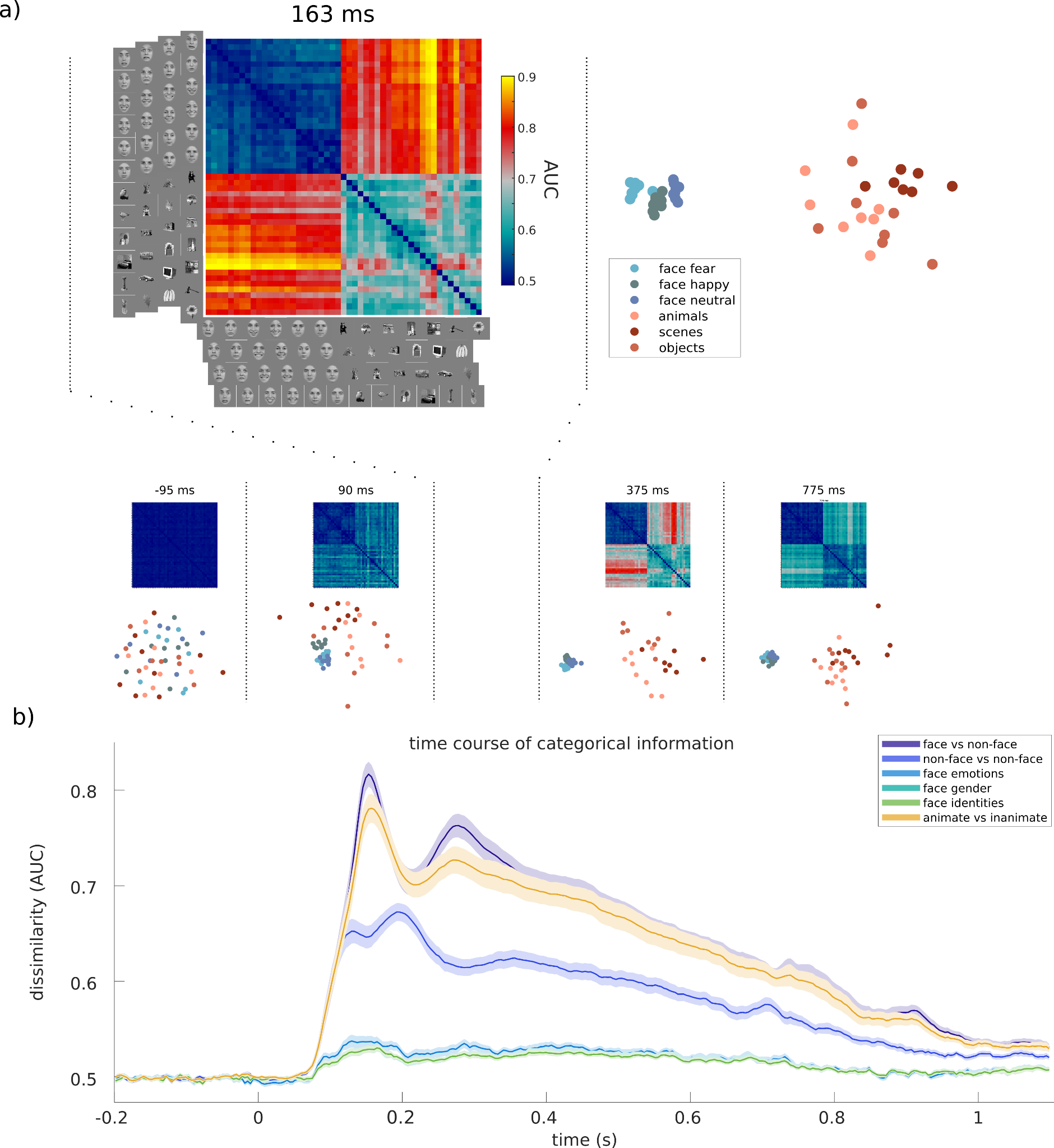
**

**Figure S4. EEG representational geometry dynamics.** a) Representational Similarity Analysis (RSA) was applied to time-resolved EEG patterns, using decoding AUC as dissimilarity measure between pairs of images (5 fold cross-validation, 5 repetitions) to create Representational Dissimilarity Matrices (RDMs). Multidimensional scaling was employed to visualise these high-dimensional brain representations on a 2D plane, which showed clear distinctions between various categories (e.g. face clusters, scenes clusters, animal clusters, etc.). b) We revealed categorical information unfolding in time by averaging dissimilarities between stimulus categories (e.g. faces vs non-face objects) and averaging across participants. Brain representations for the distinction of face vs. non-face objects (a hallmark of the N170, [(Rossion & Jacques, 2012)](https://paperpile.com/c/CVKlJ3/Kk8Nf) dominated all other categorical distinctions [(Carlson et al., 2013; Kaneshiro et al., 2015)](https://paperpile.com/c/CVKlJ3/ueTDU+usCLV), and peaked at 153 ms.

**Figure S5.** **Comparison of super- and typical-recogniser brain representations with those of CNNs.** **a**) Unconstrained mutual information for the AlexNet model showed similar effects (middle layers 3, 5) than the RDMs constrained with the semantic model (shown in figure 3). The shaded areas of all curves represent the standard error. **b**) Mutual information results comparing brain RDMs and RDMs from another similar convolutional model, VGG16, are shown for typical- (grey curve) and super-recognisers (pink curve). We found greater similarity with mid-level visual computations as indexed from this other CNN model (layers 4, 5) in the brains of super-recognisers (black line indicates significant contrasts, *p*<.05, cluster-corrected) between 133 ms to 165 ms, similarly to what is shown in figure 3 with AlexNet.
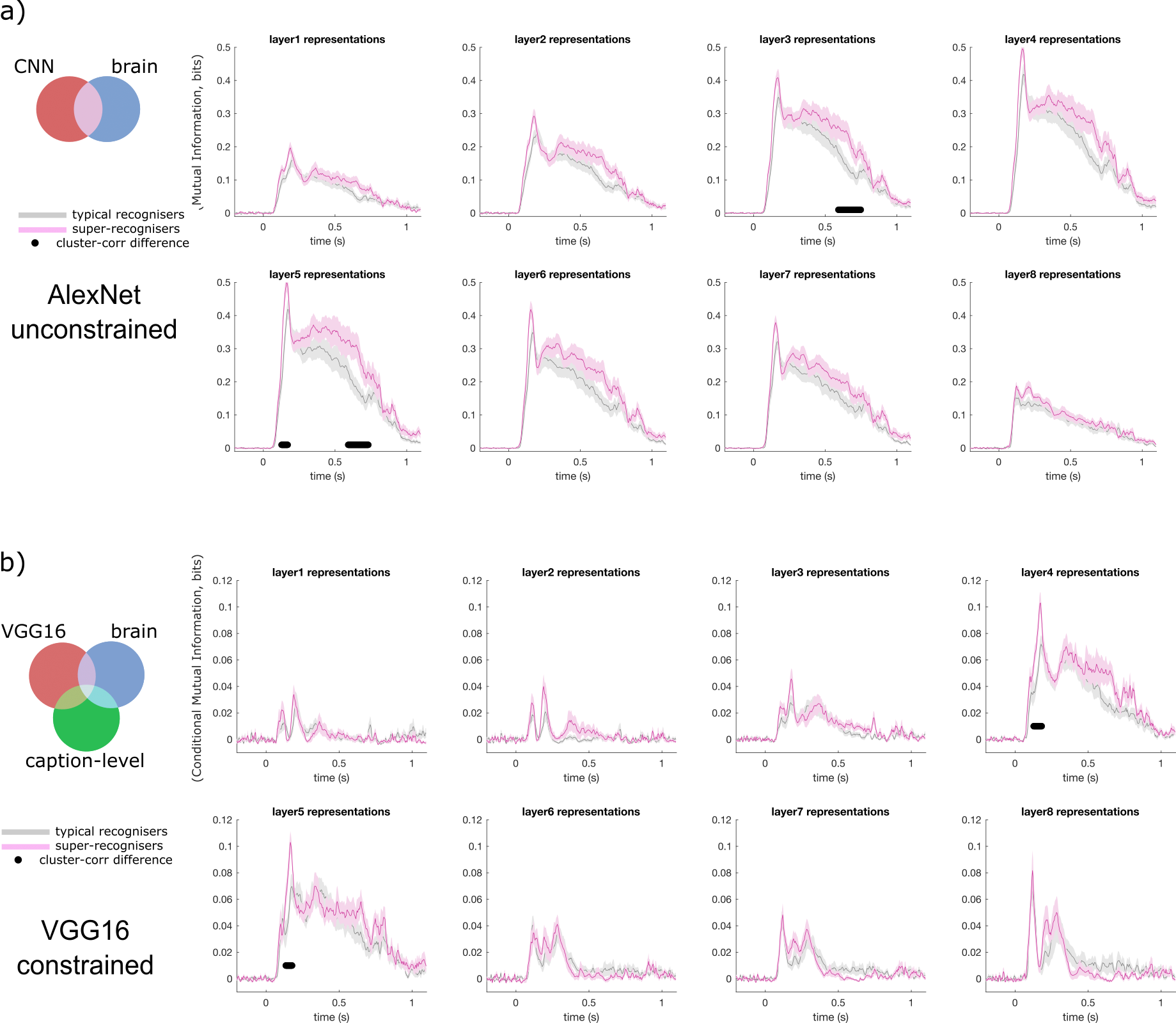


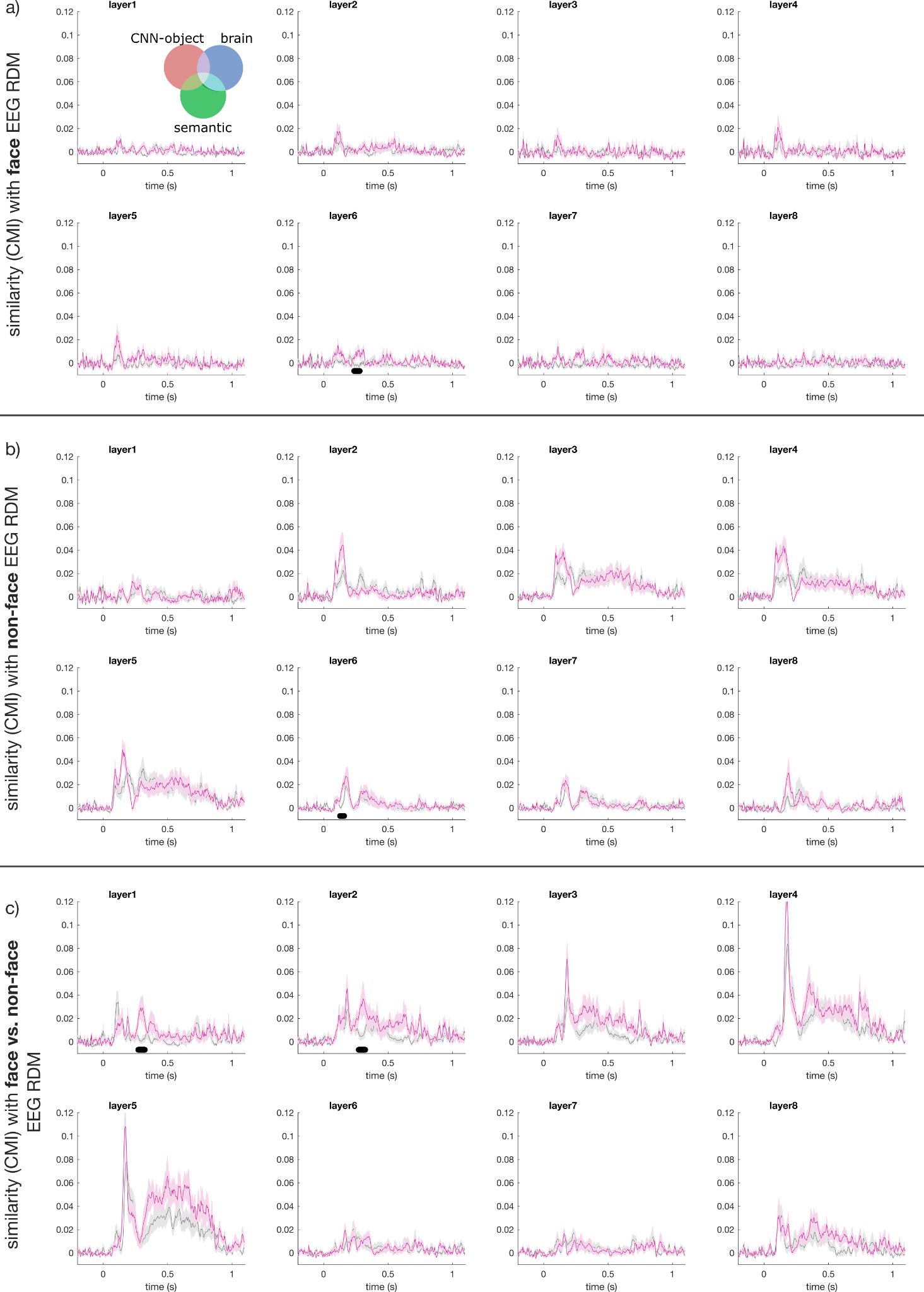


**Figure S6. Associations with object-trained AlexNet on face-only, non-face only, and non-face vs. face only RDM conditions. a)** Association between AlexNet model RDMs and brain RDMs for *face-stimuli only* across EEG time are shown for SRs (pink traces) and typical recognisers (grey traces). Significant differences between SRs and TRs peaked in the layer 6 of the artificial neural network 300 ms after face onset. **b)** The same comparison was made but using the object-only conditions of the RDMs. We observed one significant cluster between SRs and TRs in this condition. **c)** The same comparison was made but using the face vs. non-face only conditions of the RDMs. We observed two significant clusters between SRs and TRs in this condition.


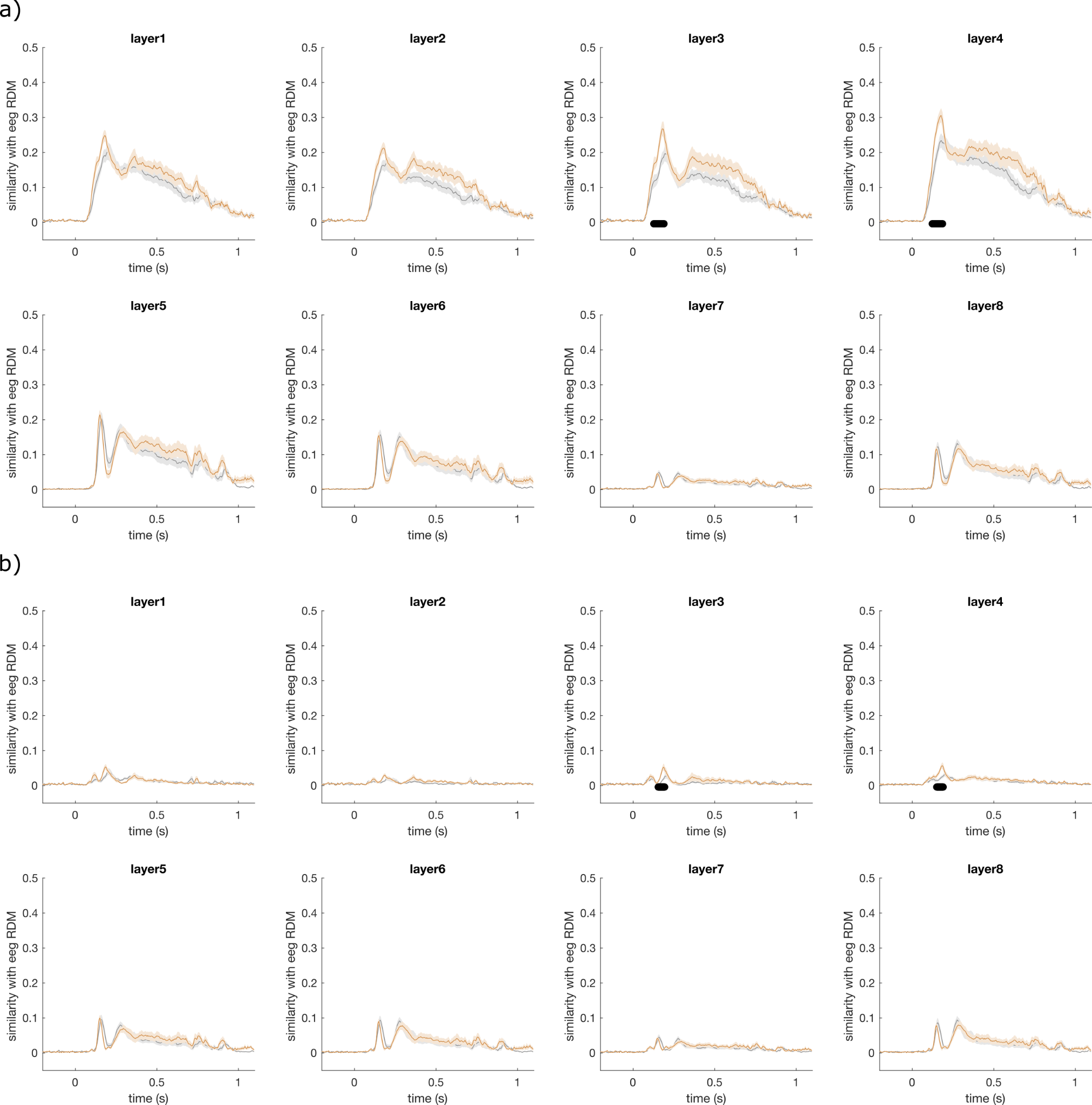


**Figure S7. Associations with face-trained (vgg-face) convolutional neural network model.** **a)** Association between face-trained VGGface model RDMs and brain RDMs for all RDM across EEG time are shown for SRs (orange traces) and typical recognisers (grey traces). Significant differences between SRs and TRs peaked in the mid-layers of the face artificial neural network (conv3_3, conv4_3) around 127 to 190 ms after image onset. **b)** Association between face-trained VGGface model RDMs and brain RDMs – constrained on the semantic model – are shown for SRs (orange traces) and typical recognisers (grey traces) across EEG time. This also revealed significant differences between SRs and TRs, peaking in the middle layers of the face-trained artificial neural network (layer 3, layer 4) around 154 to 193 ms after image onset.


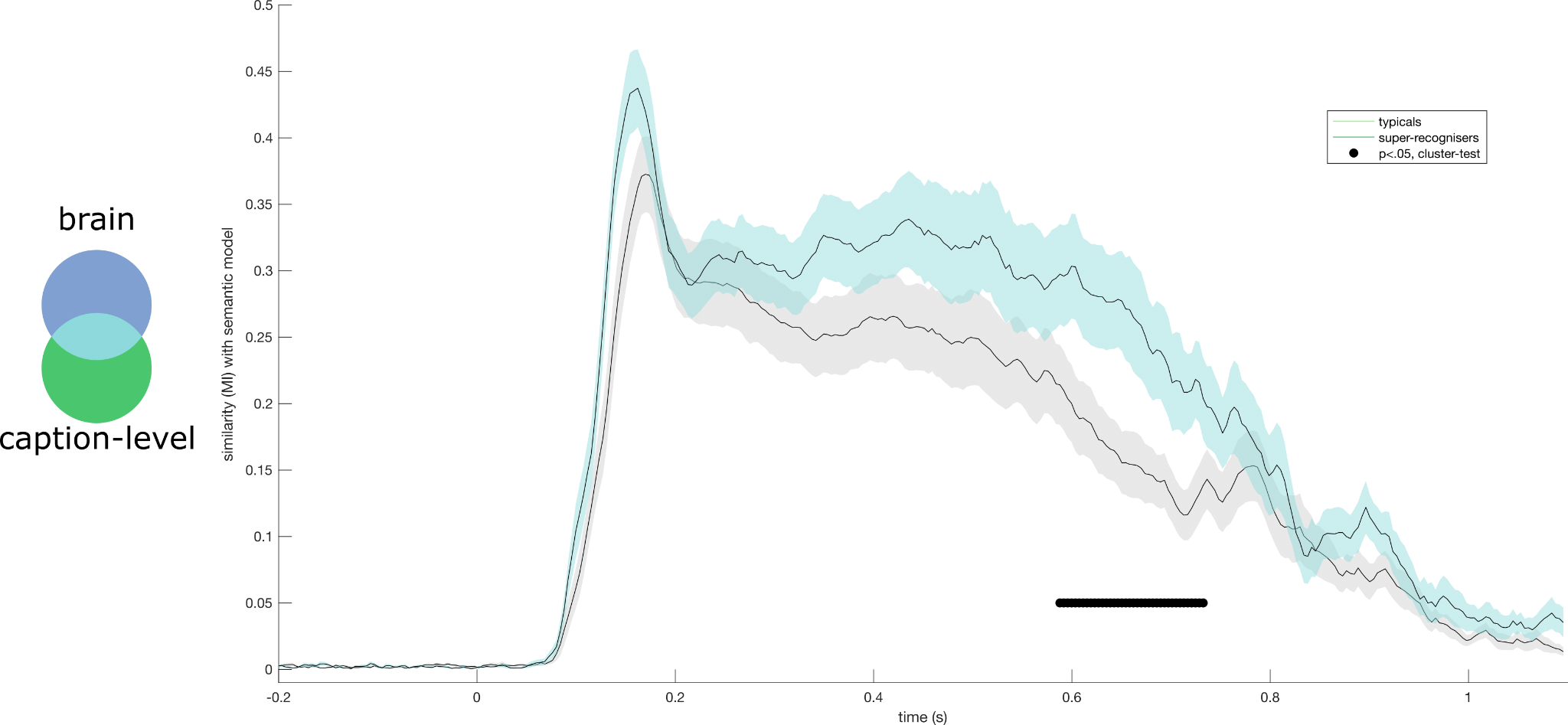


**Figure S8. Comparison of super- and typical-recogniser brain representations with those of the semantic model, unconstrained.** Unconstrained mutual information for the semantic model showed similar effects than the RDMs constrained with the CNN model (shown in figure 3).


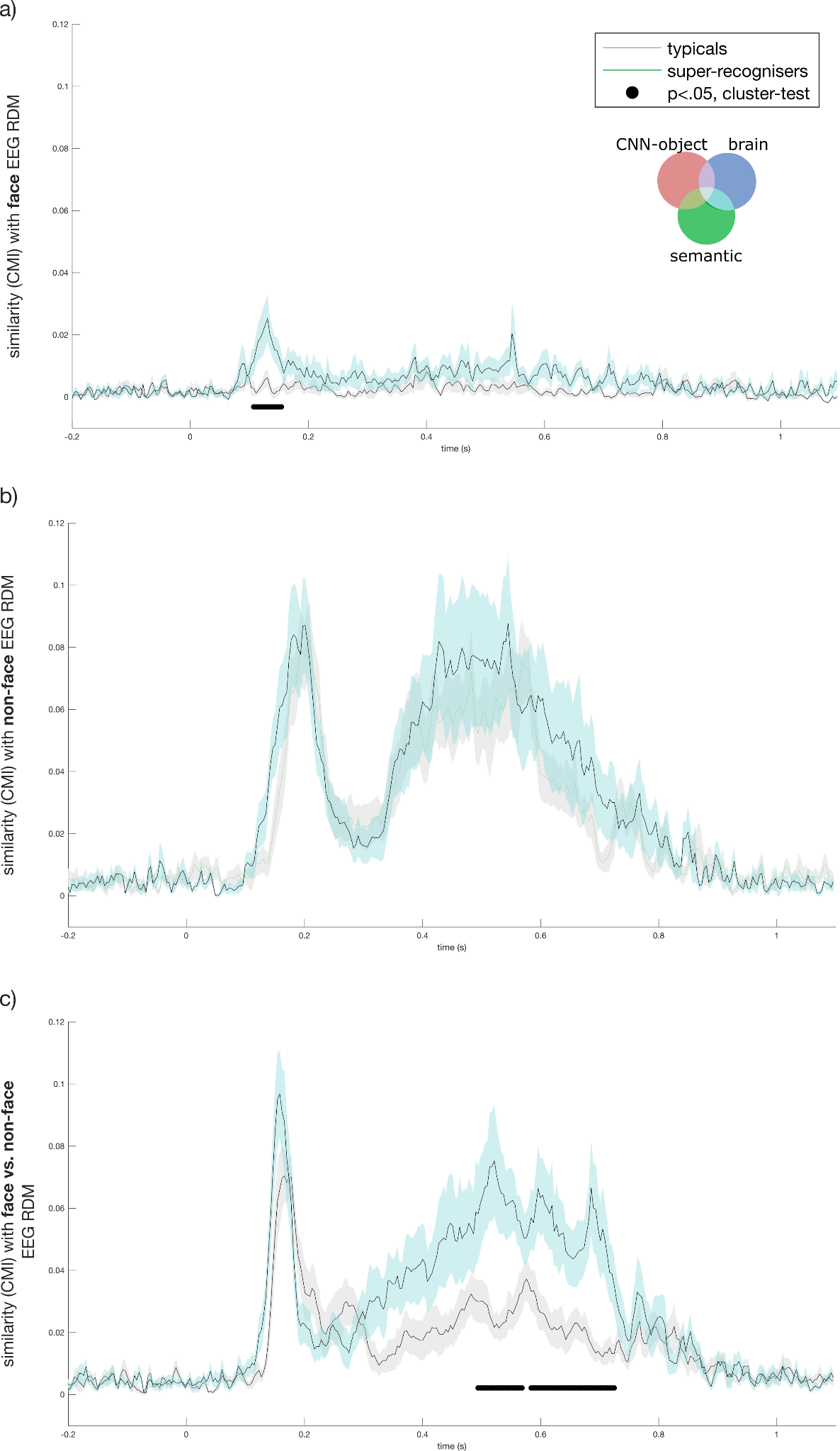


**Figure S9. Associations with semantic-trained model on face-only, non-face only, and non-face vs. face only RDM conditions. a)** Association between a semantic embedding model RDMs and brain RDMs for *face-stimuli only* across EEG time are shown for SRs (cyan traces) and typical recognisers (grey traces). Significant differences between SRs and TRs were found around 150 ms after face onset. **b)** The same comparison was made but using the object-only conditions of the RDMs. We observed no significant cluster between SRs and TRs in this condition. **c)** The same comparison was made but using the face vs. non-face only conditions of the RDMs. We observed two significant clusters between SRs and TRs around 600 ms, putatively driving the effects shown in figure 3.
